# Case of Cystocele Cured by Sims’ Operation of Elytrorrhaphy

**Published:** 1876-07

**Authors:** R. M. Lackey

**Affiliations:** Maywood, Ill.


### A CASE OF CYSTOCELE CURED BY SIMS' OPERATION OF ELYTRORRHAPHY.

By R. M. LACKEY, M.D., Maywood, Ill.

Fortunately for womankind, the disease we are about to consider is not of *very* frequent occurrence, yet it is met with often enough, and occasions sufficient suffering to demand of us the most careful study, and the best efforts for its cure.

When the anterior wall of the vagina, which adheres closely to the bladder, becomes greatly weakened and stretched from any cause, a pouch may descend into the vagina, and thus constitute a cystocele, or hernia of the bladder. This pouch may be small, and give but little discomfort to the patient, and perhaps escape entirely the notice of the surgeon, or it may be large enough to descend to and even protrude from the vulvar orifice, causing the most intense suffering. It becomes filled with urine, which cannot be thoroughly evacuated by the ordinary efforts at micturition, and the urine thus retained decomposes and becomes the source of extensive irritation, vesical catarrh, dysuria, etc.

The suffering is intensified when the patient attempts to maintain the erect position, by a sense of weight and dragging down from the umbilicus, and intolerable tenesmus. The vagina and protruded portion of the bladder become exceedingly tender, so that an effort to pass the finger, or make an examination with the speculum, may be frustrated, unless the patient be first anæsthetized. Owing to the distress occasioned by the erect position, sufferers from this malady are liable to become bedridden, especially as the disease is always complicated with some abnormal position or condition of the uterus. Prolapsus and anterior flexion are perhaps the most frequent forms of displacement met with. Ulceration, chronic inflammation and enlargement may be present, also hæmorrhoids ; these latter probably caused, or at least aggravated, by straining while attempting to void urine.

Some of the causes which operate to produce this disease are ruptured perineum, anteflexed uterus, straining, lifting heavy weights, frequent parturitions ; and, in general, those agencies which most frequently cause uterine displacements and vaginal weakness and prolapse.

It is only by careful digital examination that cystocele can be detected. In passing the finger within the vulvar orifice it comes in contact with a soft, fluctuating tumor, which can be readily pressed upward. It must be borne in mind, however, that tumors of this character may attend prolapsus of the vaginal walls in conjunction with ascites. If a cystocele be present, by pressing up the tumor the patient will be enabled to evacuate the bladder perfectly, and the urine contained in the pouch will be fetid and ropy. Should any doubt exist as to the nature of the tumor, a catheter passed into the bladder will pass downward into the vesical diverticulum, and may be felt through the vagina, thus rendering certain the character of the disease.

The measures employed for the cure of cystocele are mainly mechanical support and surgical procedures. Where the tumor is slight and without embarrassing complications, some of the various pessaries, together with astringent injections and proper hygienic management, may be all that is required to afford relief; but where the disease has reached an aggravated form, and is attended with troublesome complications, no confidence should be placed in anything, save the surgical operation employed in the case I am about to relate.

The following case presents a history of interest, running back several years. It will suffice for our present purpose, however, to give briefly its history for the two years preceding the operation:

Mrs. G., aged thirty-eight years: wife of a farmer; mother of six children, youngest three years old; she is rather above medium size; tissues flabby, and poorly nourished. She has borne children rather rapidly, and did not get up well from her last confinement; had chills, pain and tenderness of the abdomen, frequent desire to pass water, dribbling of urine, obstinate constipation, pain, heaviness and throbbing in the vagina, and dragging from the umbilicus. These symptoms were especially aggravated when she attempted to get up and go about, so that, to secure any degree of comfort, she was obliged to spend most of her time in the recumbent position. While suffering in this way, she was treated for ague, inflammation and ulceration of the womb, disease of kidneys, bladder, bowels, etc. Three or four different medical men had successively had charge of her case, without seeming to discover the true nature of her complaint, or affording her any permanent relief. At the time I first saw her she had been bedridden for more than a year, and was weak and despondent, and unwilling to submit to any further treatment. She, however, consented to an examination, and in passing my finger within the vulva, I found a soft, fluctuating tumor, the size of a hen's egg, well down between the labia majora. The hyperæsthesia of the parts was excessive. and I was compelled to desist from making a thorough examination until she was anæsthetized, which was done. When she was sufficiently under the influence of the anæsthetic I pursued my investigations further, by pressing the tumor upward so as to reach the uterus, which was found prolapsed and enlarged, and the os ulcerated. A catheter was introduced into the bladder, and it passed downward into the vesical pouch, and I was enabled to draw off a quantity of dirty, fetid urine. The vaginal mucous membrane was congested, and covered with a whitish discharge. The case was thus clearly proven to be one of cystocele, with its usual complications. Having stated the nature of the case clearly to the patient and her friends, and given some encouragement as to the success of surgical procedures, an operation was at last agreed to. Astringent applications were ordered for the vagina, and tonics and nutriment by the mouth, in the hope of improving her condition generally and locally, before proceeding further.

*Elytrorrhaphy.*—This is the elegant term employed to designate the operation for lessening the calibre of the vagina, for the cure of cystocele, prolapsus uteri et vaginæ, rectocele, etc.

It is a long while since the first attempts to perform the operation were essayed, but without complete success until the use of Sims' duck-bill speculum, and the means it affords of perfectly exposing the vaginal walls. The operation now in most common use is known as "Sims' Operation of Elytrorrhaphy," and is attended with very favorable results.

In the case here reported, the amount of tissue to be got rid of was very large, and some doubts arose as to a single operation resulting in complete relief. The patient having improved sufficiently to warrant us in proceeding, the operation was performed as follows:

She was etherized and placed in the position recommended in the operation for vesico-vaginal fistula; a curved sound with tenaculum points was fixed in the os uteri, and the walls of the vagina made to fold over it, so as to determine where union must take place. I then proceeded to denude the edges of these folds, commencing at the neck of the bladder, and extending upward as high as the os uteri. The sound was then removed, and the line of denudation continued transversely, connecting the other two, making a triangular or trowel-shaped space two inches wide at the base. The amount of hæmorrhage was inconsiderable. These denuded lines were then drawn together with fifteen interrupted silver sutures, passed from their point of entrance on one side of the freshened lines through the triangular space to the point of exit on the other side. The upper one, through the base of the space, was passed as a running stitch, so as to shut up the opening that would otherwise be left in the sack formed by this fold of the vaginal walls. This latter point is especially insisted on by Dr. J. Addis Emet, for if the opening is not thus closed, the os uteri becomes engaged in it, and it may also become filled with the discharges.

The operation was tolerably well borne. Some retching followed, and I remained with her while this lasted, and kept a sponge in the vagina to support the weak point, lest the straining should tear loose the sutures. The bladder was emptied with a catheter every five or six hours for five days, when she was then able to dispense with it. The dribbling ceased also. The bowels were kept quiet for a week, and then moved by enema. Half the sutures were removed on the fourteenth day, and the rest on the seventeenth day. On removing the last sutures the union was found to be complete, and the constriction sufficient to retain the bladder in place. The uterus was in its normal position also. The patient's general health improved rapidly, so that at the end of six weeks after the operation she was able to be up and move around with more comfort than for many months previously. The hyperæsthesia had so far disappeared that she could endure vaginal examination without taking ether.

A report from the case six months afterward shows that the improvement continued permanently ; the cure of the cystocele and its complications was complete. The patient had so far recovered that she was attending to her household duties, and had taken a journey some distance to visit friends.

The results in this case would lead us to believe that this operation should be resorted to more frequently than it is, for the radical cure of vesico-vaginal hernia, and prolapsus uteri, where other means so often prove unavailing ; and I have no doubt it would be, were it not so difficult to get the consent of the patients to operative procedures, except in extreme cases. My object in reporting this case is to place on record some additional evidence of the usefulness of the operation, and encourage its judicious employment.

